# Risk of malnutrition is associated with mental health symptoms in community living elderly men and women: The Tromsø Study

**DOI:** 10.1186/1471-244X-11-112

**Published:** 2011-07-17

**Authors:** Jan-Magnus Kvamme, Ole Grønli, Jon Florholmen, Bjarne K Jacobsen

**Affiliations:** 1 Department of Community Medicine, Faculty of Health Sciences, University of Tromsø, N-9037 Tromsø, Norway; 2 Department of Gastroenterology, University Hospital North Norway, N-9037 Tromsø, Norway; 3 Department of Geriatric Psychiatry, University Hospital North Norway, N-9037 Tromsø, Norway; 4 Laboratory of Gastroenterology and Nutrition, Institute of Clinical Medicine, University of Tromsø, N-9037 Tromsø, Norway

## Abstract

**Background:**

Little research has been done on the relationship between malnutrition and mental health in community living elderly individuals. In the present study, we aimed to assess the associations between mental health (particularly anxiety and depression) and both the risk of malnutrition and body mass index (BMI, kg/m^2^) in a large sample of elderly men and women from Tromsø, Norway.

**Methods:**

In a cross-sectional survey, with 1558 men and 1553 women aged 65 to 87 years, the risk of malnutrition was assessed by the Malnutrition Universal Screening Tool ('MUST'), and mental health was measured by the Symptoms Check List 10 (SCL-10). BMI was categorised into six groups (< 20.0, 20.0-22.4, 22.5-24.9, 25.0-27.4, 27.5-29.9, ≥ 30.0 kg/m^2^).

**Results:**

The risk of malnutrition (combining medium and high risk) was found in 5.6% of the men and 8.6% of the women. Significant mental health symptoms were reported by 3.9% of the men and 9.1% of the women. In a model adjusted for age, marital status, smoking and education, significant mental health symptoms (SCL-10 score ≥ 1.85) were positively associated with the risk of malnutrition (odds ratio 3.9 [95% CI 1.7-8.6] in men and 2.5 [95%CI 1.3-4.9] in women), the association was positive also for subthreshold mental health symptoms. For individuals with BMI < 20.0 the adjusted odds ratio for significant mental health symptoms was 2.0 [95% CI 1.0-4.0].

**Conclusions:**

Impaired mental health was strongly associated with the risk of malnutrition in community living elderly men and women and this association was also significant for subthreshold mental health symptoms.

## Background

Mental health problems are among the most prevalent conditions in elderly people. Anxiety and depression, often seen as co-morbid conditions with overlapping symptoms [1], are the two most frequent mental health disorders [2]. Malnutrition is also relatively common in elderly individuals and may be associated with mental health, particularly depression [3].

While several studies have found mental disorders to be a risk factor for involuntary weight loss/malnutrition in geriatric inpatients and outpatients [4], little population-based research has been done on the relationship between risk of malnutrition and mental health in this age group. A study from Sweden found depressive symptoms to predict malnutrition in community living elderly [5], whereas a German study of nursing home residents found no significant difference in the mean malnutrition score between residents with and without depression [6]. Furthermore, studies of the relationship between body mass index (BMI) and depressive symptoms in elderly individuals have yielded conflicting results. In a study from the US, depression in men was found to be inversely associated with body weight [7]. A later study of a multiethnic elderly population found an increased risk of depression with increasing BMI, but the most adverse impact of obesity on depression was found in African Americans [8]. Neither of these studies examined the lower BMI categories in more detail.

In the current study, we therefore aimed to investigate the associations between mental health and both the risk of malnutrition and BMI in a large sample of community-living elderly men and women. We hypothesised that there is a positive relationship between impaired mental health and risk of malnutrition and low BMI.

## Methods

### Study population

Between October 2007 and December 2008, adult inhabitants of the community of Tromsø were invited to participate in a health survey known as the Tromsø Study. In the current analysis, we included data from participants aged 65 to 87 years. All 6098 men and women in this age group were invited, and 4017 (65.9%) completed the survey. Height or weight was not measured in 21 persons and information about weight loss that was required for the determination of malnutrition was missing in 413 persons; in addition, 472 persons omitted data related to smoking, education or mental health symptoms. Therefore, 1558 men and 1553 women (51.0% of the invited individuals) were included in the analysis. The mean age of the participants included in the study sample was lower than that of the non-attending persons, and the mean age was also lower than that of the participants not included in the study sample because of missing values. The BMI of the included participants was not significantly different from that of the non-included participants.

Each participant provided written informed consent, and the survey was approved by the Regional Board of Research Ethics.

### Measures

#### Nutritional screening tool and body mass index

The participants had their weight (kg) and height (cm) measured to the nearest decimal. During these measurements, they were in light clothing and did not wear shoes. BMI was calculated as the weight divided by the square of height (kg/m^2^). In a self-administrated questionnaire, the participants were asked for any involuntary weight loss during the last six months (and if so, weight loss in kg). Weight loss was grouped as follows: below 5%, between 5% and 10% or above 10% of their pre-weight-loss body weight.

**Figure 1:**
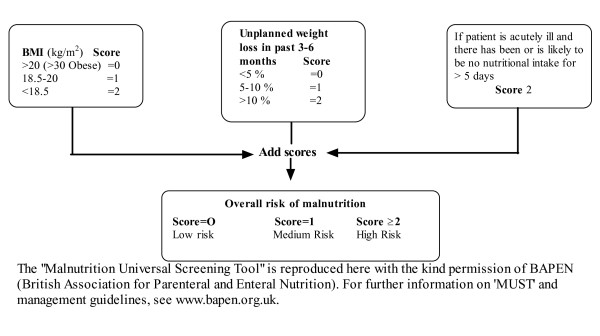
**The malnutrition universal screening tool ('MUST') is composed of a BMI score, a weight-loss score and an acute illness component**. The risk of malnutrition can be assessed based on the sum of these scores.

Based on the BMI and the extent of weight loss, each subject was categorised into low, medium or high risk of malnutrition according to the Malnutrition Universal Screening Tool ('MUST') (Figure 1). The 'MUST' tool is the nutritional screening instrument recommended by the European Society for Clinical Nutrition and Metabolism (ESPEN) for use in the community [9]. Two other nutritional screening tools have been recommended by the ESPEN, the Nutrition Risk Screening 2002 (NRS 2002) and the Mini Nutritional Assessment (MNA). NRS 2002 is mainly intended for use in hospitals. The MNA is constructed to be used by heath care professionals and not for self-administration. Consequently, the MNA is difficult to use in larger epidemiological studies.

**Figure 2:**
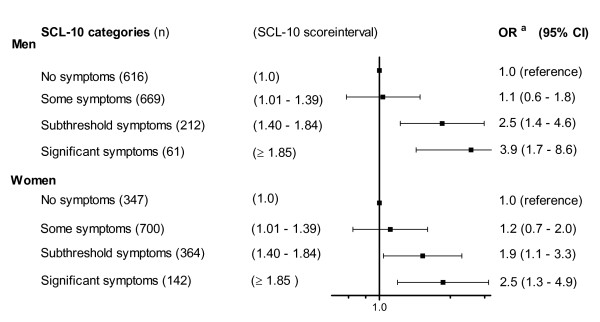
**Odds ratio for the association between mental health problems (in four categories) and the risk of malnutrition (combining medium and high risk) in 1558 elderly men and 1553 elderly women, The Tromsø Study**. ^a^Adjusted for age, smoking, marital status and educational level.

The 'MUST' tool was originally developed by the British Society of Parenteral and Enteral Nutrition http://www.bapen.org.uk. It includes an acute disease component with no nutritional intake for > 5 days, which normally necessitates hospitalisation [10]. Because participation in this study required the ability to independently visit a research centre, the acute diseases component was set to zero. The weight loss question was slightly modified to state a time span of the "last 6 months", but this encompasses the time span of "the past 3-6 months", as stated in the original 'MUST' tool. In Tables 1 and 2, all three risk categories of malnutrition are described, whereas the medium and high risk categories are combined in the analyses in Figure 2.

**Table 1:** Baseline characteristics of participating elderly men and women, The Tromsø Study (2007-2008)

	Men (*n *= 1558)	Women (*n *= 1553)	*p*-value
Age in years, Mean (SD)	71.2 (5.3)	72.0 (5.6)	< 0.001^a^
Currently married, % (n)	75.6 (1178)	51.4 (798)	< 0.001^b^
Lower education, % (n)	33.2 (517)	52.9 (822)	< 0.001^b^
Smoking, % (n)			
Never smoked	24.4 (380)	47.8 (743)	< 0.001^b^
Previous smokers	60.6 (944)	38.2 (593)	
Current smokers	15.0 (234)	14.0 (217)	
Alcohol^d ^more than once a month, % (n)	57.1 (878)	39.6 (605)	< 0.005^b^
BMI (kg/m^2^) Mean (SD)	27.0 (3.6)	27.0 (4.5)	0.69^a^
Risk of malnutrition, % (n)			
Low	94.3 (1470)	91.4 (1419)	0.005^b^
Medium	3.5 (55)	5.5 (85)	
High	2.1 (33)	3.2 (49)	
SCL-10 score Median (interquartile range)	1.10 (1.00-1.30)	1.20 (1.07-1.44)	< 0.001^c^
SCL-10 score ≥ 1.85, % (n)	3.9 (61)	9.1 (142)	< 0.001^b^

^a^t-test, ^b^chi-square test, ^c^Mann-Whitney U test, ^d ^*n *is 1538 men and 1526 women (alcohol).

**Table 2:** The SCL-10 score^a ^according to risk categories of malnutrition in elderly men and women, The Tromsø Study (2007-2008)

		Men (*n *= 1558)			Women (*n *= 1553)	
	
Risk of malnutrition	n	SCL-10 score	*p*-value^b^	n	SCL-10 score	*p*-value^b^
Low	1470	1.10 (1.0-1.30)		1419	1.20 (1.05-1.40)	
Medium	55	1.13 (1.10-1.40)	< 0.001	85	1.30 (1.10-1.65)	< 0.001
High	33	1.36 (1.05-1.56)		49	1.40 (1.13-1.70)	

^a^Median (IQ range), ^b^Kruskal Wallis test.

BMI was divided into six categories in order to include the World Health Organization definitions of overweight (25.0-29.9 kg/m^2^) and obesity (≥ 30 kg/m^2^) [11] in addition to the underweight category (< 20 kg/m^2^) [9]. We further subdivided the categories between 20 kg/m^2 ^and 30 kg/m^2 ^to describe in more detail the lower-normal weight (20.0-22.4 kg/m^2^, 22.5-24.9 kg/m^2^) and overweight individuals (25.0-27.4 kg/m^2^, 27.5-29.9 kg/m^2^).

#### Assessment of mental health symptoms

Mental health status was assessed by the Hopkins Symptoms Check List-10 (SCL-10), which has been widely used in epidemiological studies. The SCL-10 is a self-administrated instrument that mainly explores symptoms of anxiety and depression [12]. The ten items of the SCL-10 were part of the questionnaire that was included in the invitation to the survey. The questionnaire was completed by participants at home and handed in at the study centre.

The SCL-10 questions explored the presence and severity of the following ten symptoms during the preceding week: (1) "Sudden fear without apparent reason", (2) "Afraid or worried", (3) "Faintness or dizziness", (4) "Tense or upset", (5) "Easily blaming yourself ", (6) "Sleeplessness", (7) "Depressed or sad", (8) "Feeling worthless", (9) "Feeling that everything is a struggle", and (10) "Feeling hopelessness with regard to the future".

Each question was rated on a four-point scale ranging from 1 (not at all) to 4 (extremely). Missing values were replaced by the sample mean value for each item, but questionnaires with three or more missing values were excluded from the analyses. The average SCL-10 score was calculated according to Strand et al [12] by dividing the total score by the total number of items (score ranging between 1.0 and 4.0). A higher score value indicated more symptoms. We found an acceptable degree of internal consistency for the scale in this sample (Cronbach's alpha 0.84).

The SCL-10 is an abbreviated version of the 25-item Hopkins Symptoms Checklist (SCL-25) [13], which has been validated in different age categories, including elderly individuals [14]. The SCL-25 was designed to predict both anxiety and depression but was found to predict depression better than anxiety disorders in a population-based study [15]. The shorter SCL-10 version correlated highly with the SCL-25 version (r = 0.97) in a population-based Norwegian study that also included elderly individuals [12]. Depending on the cut-off limits used, the literature indicates that 50-60% of cases detected with these instruments are individuals who actually qualify for a diagnosis of mental disorders based on clinical interviews [12].

An SCL-10 score of 1.85 has been proposed as the cut-off for predicting diagnosed mental disorders [12], and score values of ≥ 1.85 in the current study were referred to as *significant symptoms*. To assess the impact of score values below this cut-off, we subdivided the SCL-10 scores between 1.01 and 1.84 into a lower score category (SCL-10 score 1.01 to 1.39) referred to as *some symptoms *and a higher score category (SCL-10 score 1.40 to 1.84) referred to as *subthreshold symptoms*. The individuals with no symptoms (SCL-10 score 1.0) constituted the reference category (Figure 2).

#### Other variables

Information regarding age and marital status was obtained from Statistics, Norway. Details regarding educational background, household income, smoking habits and other disease variables were obtained from self-administrated questionnaires. Household income was dichotomised into above and below Norwegian Kroner 300 000. Lower education was defined as primary school only. Alcohol use was relatively infrequent and was dichotomised into drinking more than once a month versus a lower consumption. Smoking habits were divided into three categories (never, previous or current smoking).

### Data analysis

The SCL-10 score was analysed as both a dichotomised variable and a continuous variable. The score was positively skewed and we therefore reported the median SCL-10 values with 25 - 75% interquartile (IQ) range in Tables 1 and 2. The Mann Whitney U or Kruskal Wallis test was used to test the differences in SCL-10 score between the groups. Differences in baseline variables between men and women were analysed using the Chi-square test and t-test (Table 1). The associations between the SCL-10 categories and the risk of malnutrition were analysed using logistic regression (Figure 2). The SCL-10 category with no symptoms (1.0) was used as reference. The odds ratio (OR) estimates were adjusted for potential confounders (age, marital status, smoking and educational level). The analysis of the relationship between the risk of malnutrition and the SCL-10 score was stratified by gender. The Chi-square test and logistic regression (table 3) were used to analyse the relationship between the six BMI categories and the proportion of the participants with an SCL-10 score ≥ 1.85. In the regression analysis, the BMI category with the highest number of participants was used as reference. Data from men and women were pooled in this analysis due to the low expected numbers in some BMI groups in sex-stratified analyses.

**Table 3:** The proportion of subjects with SCL-10 score ≥ 1.85 and odds ratio (95% confidence interval) for the association between SCL-10 score ≥ 1.85 and BMI in elderly men and women^b^, The Tromsø study (2007-2008)

BMI categories	SCL-10 score ≥ 1.85 % (proportions)	OR (95% CI) for SCL-10 score ≥ 1.85	
		**Adjusted for** **age and sex**	**Multivariable** **adjusted^a^**

< 20.0	15.2 (12/79)	2.3 (1.1-4.5)	2.0 (1.0-4.0)
20.0-22.4	5.2 (16/308)	0.8 (0.4-1.4)	0.8 (0.4-1.4)
22.5-24.9	6.7 (42/631)	1.1 (0.7-1.8)	1.1 (0.7-1.7)
25.0-27.4	5.6 (45/803)	1.0 Reference	1.0 Reference
27.5-29.9	6.5 (42/646)	1.1 (0.7-1.8)	1.1 (0.7-1.7)
≥ 30.0	7.1 (46/644)	1.2 (0.8-1.8)	1.2 (0.8-1.9)

^a ^Adjusted for sex, age, educational level, marital status and smoking status, ^b^*n *= 3111.

Two sided *p*-values < 0.05 were considered statistically significant. The analyses were performed using SPSS statistical software version 17.0 (SPSS inc., Chicago, Illinois, USA).

## Results

Baseline characteristics of the 1558 men and 1553 women included in the analyses are shown in Table 1. The mean age was 71.2 years in men and 72.0 years in women. Compared to men, women were more likely to be single and have a lower level of education, and a smaller proportion had a history of smoking. Mean BMI was 27.0 kg/m^2 ^in both genders. Risk of malnutrition (combining medium and high risk) was found in 7.1% (222/3112) of the participants, which included 5.6% (88/1558) of men and 8.6% (134/1553) of women. The SCL-10 score was higher in women (median 1.20) than in men (median 1.10) (*p *< 0.001) and was higher in persons aged ≥ 75 years old than in persons aged 65 to 74 years old, which indicates more symptoms of anxiety and depression in women and in the oldest participants. Significant mental health problems (SCL-10 score ≥ 1.85) were found in 3.9% (61/1558) of men and 9.1% (142/1553) of women.

### Mental health and the risk of malnutrition

The SCL-10 score was significantly associated with an increased risk of malnutrition in both men and women (Table 2). The results suggest a relatively stronger relationship between the risk of malnutrition and the median SCL-10 score in men than in women.

In men who were at risk of malnutrition (combining medium and high risk), 11.4% (10/88) had significant SCL-10 symptoms; the corresponding percentage in women was 16.4% (22/134). In Figure 2, the strength of the associations between the SCL-10 score categories and the risk of malnutrition is further explored using a logistic regression analysis. In both men and women, significant SCL-10 symptoms were strongly associated with the risk of malnutrition; the odds ratio was 3.9 (95% CI 1.7-8.6) in men and 2.5 (95% CI 1.3-4.9) in women. Also, for the subthreshold symptoms (SCL-10 score 1.40 to 1.84), a statistically significant association with the risk of malnutrition was found. A test for linear trends across the SCL-10 score categories was statistically significant for both genders (*p *< 0.001 in men and p = 0.01 in women). However, the difference between the genders with regard to the strength of the relationship (Figure 2) was not statistically significant (*p *= 0.4). The odds ratio estimates were adjusted for age, marital status, smoking habits and educational level. Individuals reporting no SCL-10 symptoms (score 1) constituted the reference category.

In three separate sets of analyses, we also adjusted for the impact of alcohol use (more or less frequent than once a month), chronic somatic diseases (history of cancer, heart attack or stroke) or household economy. However, none of these three variables had a significant impact on the relationship between the SCL-10 score and the risk of malnutrition (data not shown).

### Mental health and BMI

We also assessed the relationship between various BMI categories and the proportion of individuals (men and women) with significant SCL-10 symptoms (SCL-10 score ≥ 1.85). The highest proportion with significant SCL-10 symptoms (15.2%, 12/79) was found in participants with BMI < 20.0 kg/m^2 ^(Table 3). In obese participants (BMI ≥ 30.0 kg/m^2^) the corresponding proportion was not significantly increased. A chi-square test for the model was statistically significant (p = 0.03).

The strength of the associations between the BMI categories and a SCL-10 score ≥ 1.85 is further explored using a logistic regression analysis (Table 3). The multivariable adjusted odds ratio estimate for the lowest BMI category (< 20.0 kg/m^2^) was 2.0 (95% CI 1.0-4.0) compared to the reference category of BMI 25-27.4 kg/m^2^.

## Discussion

In this study, we found that mental health symptoms were strongly associated with the risk of malnutrition in elderly individuals. Both the risk of malnutrition and mental health symptoms were more prevalent in women than in men. To our knowledge, this is the largest population-based study that explored the relationship between the risk of malnutrition and mental health in elderly individuals.

Some previous studies in this area have utilised the Geriatric Depression Scale (GDS) and the Mini Nutritional Assessment (MNA) instrument for the assessment of the relationship between depression and malnutrition. A Swedish study of 579 community-living elderly people found that depressive symptoms were predictive of malnutrition [5]; this was observed to a larger extent in men than in women. The relationship between depression and malnutrition in nursing home residents was investigated in a German study, and no differences was found in the mean MNA score between subjects who had depression and those who did not. However, a modest association was demonstrated between malnutrition and depression in a regression analysis [6]. A study of 267 community-living elderly in Brazil [16] showed a positive relationship between malnutrition and depression.

We believe the 'MUST' tool used in the current study has an advantage over the MNA with regards to the associations explored. The MNA has been validated in a number of studies of elderly individuals, but it includes information about both neuropsychological problems and psychological stress [17]. A positive correlation between the MNA risk score and the symptoms of depression could therefore be anticipated. The 'MUST' tool does not include any component that explores mental health. This is the first study to use either the 'MUST' tool or the SCL-10 the assessment of the relationship between risk of malnutrition and mental health.

Increased risk of malnutrition (combining medium and high risk) was found in 7.1% of the individuals in the current sample. In previous studies of community-living elderly individuals, prevalence rates for the risk of malnutrition varied from 2.5% to 21% [18**-**21]. This variation in prevalence may reflect the use of different criteria both to define malnutrition and differences in sample selections.

In accordance with former studies on adult and elderly individuals, we found that women had more mental health symptoms than men [22]. This gender difference is not fully understood but may to some extent be explained by an underreporting of depressive symptoms by male individuals [23].

Mental health may be assessed by both a categorical approach, which considers diagnoses that are based on a distinct cut-off, and a dimensional approach, which considers symptoms along a continuum. The latter approach also takes into account subthreshold symptoms of anxiety and depression, which may also adversely affect daily life [24,25]. The present study revealed statistically significant associations using both a categorical and a more dimensional approach.

Somatic diseases, especially stroke, myocardial infarction and cancer, represent risk factors for depressive symptoms in elderly individuals [26]. Somatic diseases may also increase the risk of malnutrition [21]. However, adjusting for the history of these three important somatic diseases did not affect the conclusions of the current study.

Individuals with BMI < 20.0 kg/m^2 ^had a two to three times higher prevalence of significant mental health symptoms (table 3) and the corresponding adjusted OR was 2.0 and of borderline significance (p = 0.06) (table 3). Obesity (BMI >30.0) was not associated with more mental health symptoms. Previous studies have reported both a decreased [7] and an increased risk [8] of depression in obese elderly individuals. However, the lower BMI categories were not specifically examined in these two studies.

The Tromsø study included participants from both urban and rural areas although the majority live in the city centre. Our results may not be generalised to all other elderly populations as both living conditions and health care organisation differ between countries. However, we believe that it is likely that similar relationships are present in other similar community living elderly Western populations.

As discussed above, this study has several strengths as well as some potential limitations. First, the SCL-10 captures symptoms of both anxiety and depression, although depression is more influential in the relationship with nutritional status. However, considerable overlap exists between anxiety and depression, which often appear as co-morbid disorders [1,27].

Second, eating disorders were not assessed in this study. In a recent review of eating disorders in the elderly, depression was described as the most important co morbid condition. However, the prevalence of eating disorders is low in the elderly population [28].

Third, the study sample that exhibited valid values for the SCL-10-score and the 'MUST' score represented 52% of the target population. Thus, selection bias may be a concern. However, it is likely that the elderly men and women who did not complete the survey or omitted key information were frailer, more cognitive impaired and more prone to both malnutrition and impaired mental health than the persons who were included in the study sample.

Fourth, by using 1.85 as the cut-off for the SCL-10 score yielded significant mental health problems of 4.2% in men and 9.8% in women, which may be an underestimation. In elderly people, the prevalence of major depression is 1 to 4%, the prevalence of minor depression is 4 to 13% [26] and the prevalence of anxiety is 3.2% to 14.2% [29]. The cut-off of 1.85 for the SCL-10 score was adopted from previous studies that describe the SCL-10 [12] and has not been compared to clinical diagnostic interviews in community-living elderly men or women. However, the main purpose of the current study was not to describe the prevalence of mental health problems but to determine the relationship between impaired mental health and nutritional status.

Fifth, there was no screening of cognitive decline in this study. Mild cognitive impairment can be present a long time before dementia is identified and this might be associated with malnutrition and symptoms of anxiety and depression. However, participants had to both independently visit a research centre and accomplish a detailed self administrated questionnaire. This reduces the risk of cognitive impairment among participants included in the study population.

The current study also demonstrated a significant association between subthreshold mental health symptoms and the risk of malnutrition. Several reports have described other adverse health effects that are related to subthreshold mental health symptoms in elderly individuals [30,31]. The cut-off for the SCL-10 used in the current study identified 13.6% of men and 22.4% of women with subthreshold symptoms. This corresponds well with the 20.2% of older women identified with subthreshold depression in a recent study that used the Center for Epidemiological Studies Scale for Depression (CES-D) [32].

The cross-sectional design hampers conclusions about the directionality of the associations. The most important is probably the influence of depression on appetite and food intake. This can lead to weight loss and increase the risk of malnutrition. In the Diagnostic and Statistical Manual of Mental Disorders [33], both weight gain and weight loss are among the diagnostic criteria for depression. In contrast, malnutrition may also be associated with micronutrient deficiencies that adversely affect mental health. Inadequate intake of nutrients and energy may lead to deficiency of folic acid, thiamine or cobalamin [34] which might worsen mental health symptoms. A recent study that evaluated the impact of weight change alone in elderly people found that weight loss predicted an increase in depressive symptoms [35]. Hence, a bidirectional relationship between the risk of malnutrition and mental health symptoms may be present and result in a vicious circle over time in affected individuals.

## Conclusions

Impaired mental health was strongly associated with the risk of malnutrition in community living elderly men and women and this association was also significant for subthreshold mental health symptoms. For the clinical practitioner, our results on the one hand highlight the need for nutritional screening of elderly people presenting with mental health symptoms. Both in somatic and psychiatric settings, nutrition have often been neglected [3,36]. Screening for malnutrition can easily be performed by the use of instruments like the 'MUST' tool. On the other hand, mental health symptoms should also be included in the assessment of elderly people who are at risk of malnutrition.

## Abbreviations

BMI: Body Mass Index; IQ: interquartile; MUST: Malnutrition Universal Screening Tool; OR: odds ratio; SCL-10: Symptoms Check List 10.

## Conflict of interests

The authors declare that they have no conflicts of interests.

## Authors' contributions

JMK, JF, OG and BKJ were responsible for the initial design of the study. JMK did the analyses and wrote the first draft of the paper. BKJ contributed to the analyses, interpretation of the results and the review of the drafts. All authors contributed to the interpretation of the data and review of the manuscript for important intellectual content. All authors read and approved the final manuscript.

## Pre-publication history

The pre-publication history for this paper can be accessed here:

http://www.biomedcentral.com/1471-244X/11/112/prepub
